# CO_2_ Adsorption in Metal-Organic Framework Mg-MOF-74: Effects of Inter-Crystalline Space

**DOI:** 10.3390/nano10112274

**Published:** 2020-11-17

**Authors:** Siddharth Gautam, David Cole

**Affiliations:** School of Earth Sciences, The Ohio State University, 275 Mendenhall Laboratory, 125 S Oval Mall, Columbus, OH 43210, USA; cole.618@osu.edu

**Keywords:** CO_2_ storage, adsorption, metal-organic frameworks, crystallite-size, Monte Carlo simulations, inter-crystalline space

## Abstract

Metal-Organic Frameworks (MOF) have been identified as highly efficient nanoporous adsorbents for CO_2_ storage. In particular, Mg-MOF-74 has been shown to promise exceptionally high CO_2_ sorption. Although several studies have reported adsorption isotherms of CO_2_ in Mg-MOF-74, the effect of inter-crystalline spacing in Mg-MOF-74 on the sorption of CO_2_ has not been addressed. These effects have been shown to be profound for a quadrupolar molecule like CO_2_ in the case of silicalite (Phys. Chem. Chem. Phys. 22 (2020) 13951). Here, we report the effects of inter-crystalline spacing on the adsorption of CO_2_ in Mg-MOF-74, studied using grand canonical Monte Carlo (GCMC) simulations. The inter-crystalline spacing is found to enhance adsorption at the crystallite surfaces. Larger inter-crystalline spacing up to twice the kinetic diameter of CO_2_ results in higher adsorption and larger crystallite sizes suppress adsorption. Magnitudes of the inter-crystalline space relative to the kinetic diameter of the adsorbed fluid and the surface to volume ratio of the adsorbent crystallites are found to be important factors determining the adsorption amounts. The results of this study suggest that the ideal Mg-MOF-74 sample for CO_2_ storage applications should have smaller crystallites separated from each other with an inter-crystalline space of approximately twice the kinetic diameter of CO_2_.

## 1. Introduction

Over the past two centuries, atmospheric CO_2_ abundance has risen substantially [1]. As CO_2_ is a major contributor to the total energy balance that is resulting in a rise in the Earth’s temperature [2], the rise in CO_2_ abundance in the atmosphere is a cause of concern. Several strategies have been proposed to contain this rise and include, among others, CO_2_ capture and storage [3,4]. Nano-porous adsorbent materials, due to their high surface areas, can store a large amount of CO_2_ [5]. Prominent among these nano-porous materials are zeolites [6], and metal-organic frameworks (MOF) [7]. In addition to a large surface area, MOFs offer strong interaction of CO_2_ with open metal sites, thereby enhancing the adsorption amounts. In particular, Mg-MOF-74 has been found to adsorb large amounts of CO_2_ especially at low partial pressures, a condition relevant to possible applications in selectively adsorbing CO_2_ from a mixture in flue gas [8]. For this reason, several computational as well as experimental studies have been carried out to investigate various aspects of CO_2_ in Mg-MOF-74 [9,10,11,12,13]. 

In an experiment of adsorption of a fluid on a nano-porous material, the adsorbent normally consists of a large number of crystallites forming particle grains. These particle grains in turn are separated from each other and give a powder sample the grainy appearance. As a result of this, a substantial amount of empty space exists in a powder sample of an adsorbent in addition to the pores in each crystallite. The effects of this extra-crystalline space on the structural and dynamical properties of sorbed fluid have been reported in several systems [14,15,16]. In a recent computational study [17] on the adsorption of ethane and CO_2_ in silicalite, it was found that the inter-crystalline space can profoundly affect the adsorption behavior. Further, it was found that these effects are more important for CO_2_ as compared to ethane. This is possibly due to the quadrupole moment of CO_2_ that makes its interaction with the substrate stronger than that of ethane. The quadrupolar nature of CO_2_ also results in a stronger adsorption of CO_2_ compared to hydrocarbons [18,19,20,21,22] and facilitates the separation of CO_2_ from a mixture with hydrocarbons [23,24]. Because of this selective adsorption, CO_2_ can also enhance the motion of confined hydrocarbons [25,26,27,28]. 

To the best of our knowledge, no systematic study, computational or experimental, on the effects of inter-crystalline spaces on the adsorption of CO_2_ in Mg-MOF-74 has been reported. Given the importance of adsorption of CO_2_ in Mg-MOF-74, a study of the effects of inter-crystalline space on this system is needed. With this objective, we report here grand canonical Monte Carlo (GCMC) simulation studies on the adsorption of CO_2_ in Mg-MOF-74. In Section 2, we provide details of the simulations. Results from the study are presented in Section 3 while their implications are discussed in Section 4. Major conclusions drawn from the study are listed in Section 5. 

## 2. Materials and Methods 

**Figure 1:**
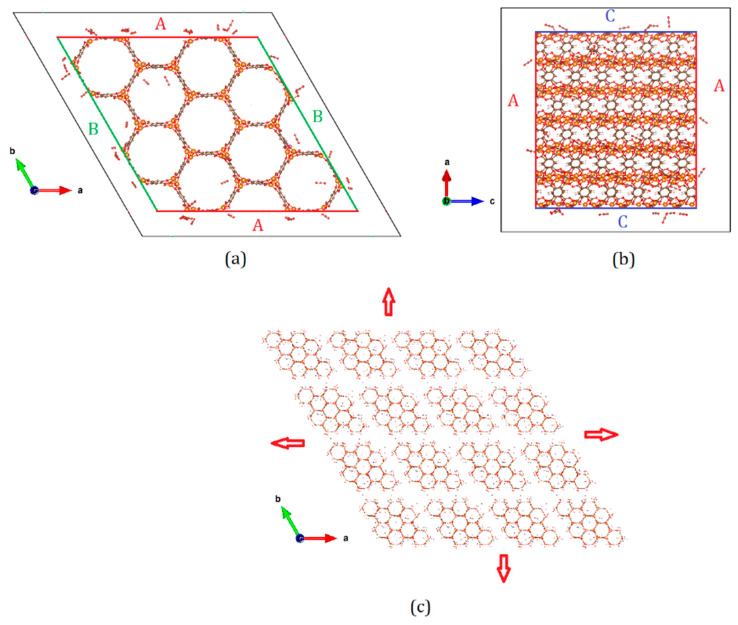
(**a**) Simulation cell in the system S4 in the X-Y plane. Simulation cell boundary is marked by the black border lines, while the borders of the supercell made by replicating a unit cell of Mg-MOF-74 2 × 2 × 6 times along the cell vectors a and b are marked by red and green lines, respectively. The colored lines thus enclose a crystallite while the space between the black and colored lines is the inter-crystalline space. Some CO_2_ molecules adsorbed within the crystallite as well as in the inter-crystalline space can be seen. (**b**) The simulation cell in the S4 system in the X-Z plane. As in (**a**) colored and black lines mark the boundaries of the crystallite and the simulation cell, respectively. (**c**) A rendering of the simulation cell in S4 in the X-Y plane with periodic boundary conditions applied. Crystallites separated by inter-crystalline space can be seen. The red arrows indicate that the system is repeated in all directions infinitely.

All simulations reported in this study were carried out using DL_Monte [29]. Unit cell coordinates of Mg-MOF-74 have been optimized by Yazgir et al. [8] and a cif file of this optimized cell was obtained from the repository [30] of the software package RASPA developed by an international collaboration between the University of Amsterdam and Delft University of Technology from the Netherlands, Universidad Pablo de Olavide, Seville, Spain and Northwestern University, Evanston, IL, USA [31]. They were used without further refinement. A single unit cell was replicated 2 × 2 × 6 times using the visualization software Vesta developed by Momma and Izumi, Ibaraki, Japan [32]. This simulation cell is designated as S0 (0 denoting the absence of any inter-crystalline spacing) and constitutes a crystallite. Further simulation cells were obtained by leaving an empty space of 1.5, 2.5, 5.0, or 7.5 Å on all sides of S0 to obtain 4 different simulation cells. These are designated S1, S2, S3, and S4, respectively. A representative snapshot from the simulation of CO_2_ adsorption in S4 is shown in Figure 1a,b. The simulation cell S0 or the crystallite can be seen within the colored boundaries in Figure 1a,b, while the boundary of the simulation cell S4 is marked with black lines (S4 = S0 + empty space). Surfaces of the crystallite parallel to the cell vectors ***a***, ***b,*** and ***c***, respectively named A, B, and C, can also be identified. With periodic boundary conditions applied in all directions, these simulation cells thus represent an infinite number of crystallites separated from each other by inter-crystalline spaces of 3, 5, 10, or 15 Å, respectively, as can be seen in Figure 1c for the representative case of S4. Results reported below indicate that S1 and S2 progressively exhibited significantly higher amounts of CO_2_ compared to S0, whereas further widening of the inter-crystalline space in S3 and S4 resulted in only marginal increase in the adsorbed amount. For this reason, additional simulations on different simulation cells with the same inter-crystalline space as in S2 were carried out to investigate the effects of variable surfaces and crystallite size. To investigate the difference in the behavior of different surfaces of S0 exposed, we prepared three simulation cells with empty spacing only on two of the six surfaces while the other four surfaces had no empty spacings. In other words, only two surfaces at a time were exposed to the inter-crystalline space in these systems. These are named A2, B2 and C2 according to the surface that is exposed to the inter-crystalline space (see Figure 1). Finally to probe the effect of crystallite size, we prepared both a small and large supercell using 1 × 1 × 4 and 3 × 3 × 9 unit cells, respectively, with empty spacing of 2.5 Å on all sides (resulting in an inter-crystalline spacing of 5 Å). These are labelled as T2 (small/tiny) and L2 (large), respectively. A summary of all the simulation cells used in this study is provided in Table 1.

The initial configuration considered one CO_2_ molecule placed in all the simulation cells in a pore. During the simulation, the guest molecules, i.e., CO_2_, could be inserted/deleted, translated, or rotated with respective probabilities of 0.5, 0.25, and 0.25, while all Mg-MOF-74 atoms were kept rigid. Although use of flexible MOF in the simulations has been found to result in more accurate adsorption isotherms, the flexible framework is found to have a significant effect only for tight-fitting larger molecules, while for a small molecule like CO_2_ a rigid framework can provide reasonable accuracy [33]. All simulations were carried out using a series of gas partial pressures (up to 100 atm) at 298 K. In DL_Monte, it is possible to directly use partial pressure of the gas instead of chemical potential as the imposed quantity. This is done by using the following selection procedures for the insertion (P_i_) or deletion (P_d_) of a molecule.

P_i_ = min{1,(βVP)exp(−βΔU)/(N + 1)} (1)

P_d_ = min{1,(N/βVP)exp(−βΔU)} (2)

where V is the simulation cell volume, P is the partial pressure of the gas, U represents the potential energy, N is the number of molecules and β = (k_B_T)^−1^; k_B_ being the Boltzmann constant. To investigate possible temperature dependence in the effects of inter-crystalline space on the adsorption of CO_2_ additional simulations at 0.1, 1.0, and 10.0 atm partial pressures were carried out at 278, 288, and 308 K for S0, S2, and S4. CO_2_ was modeled with the TraPPE-UA force field [34], while the force field and partial charges identified by Yazgir et al. [8] were used to model Mg-MOF-74. This latter force field in turn employed a combination of UFF [35] and DREIDIG [36] force fields. All cross-term interactions were calculated using the Lorentz–Berthelot mixing rules [37]. Following the recommendation for TraPPE force field as used in this work, we have used a cut-off radius of 14 Å. The long-range electrostatic interactions were calculated using the Ewald sum method [37]. Each simulation used 2 million Monte Carlo steps, out of which the first 500,000 steps were discarded to ensure the best values at equilibrium. Coordinates were sampled every 10,000 steps. The total number of CO_2_ molecules adsorbed in the simulation cell at a given partial pressure were recorded after every 10,000 MC steps, as a result of which a large data for the number adsorbed molecules were obtained over the 2 million steps after discarding the first 500,000 MC steps for equilibration. The number of adsorbed molecules (N_ads_) at a given pressure as reported here was calculated from this data set by averaging over the 150 recorded values. The uncertainty in this was estimated as the standard deviation in the value of N_ads_. The symbol size in all the figures reported in the results section is either equal to or larger than the uncertainties in the data points.

## 3. Results

### 3.1. Effects of the Size of Inter-Crystalline Spacing

**Figure 2:**
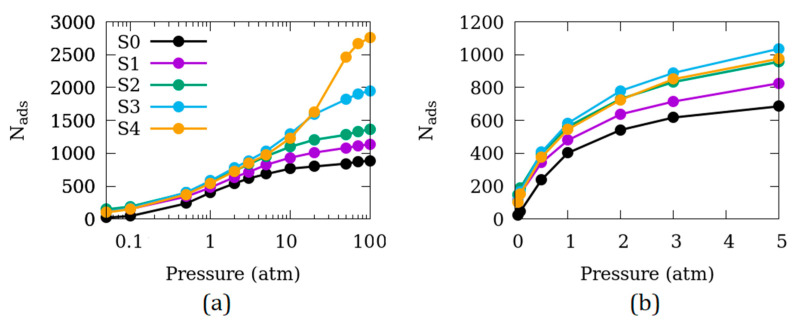
Adsorption isotherms of CO_2_ in Mg-MOF-74 over (**a**) wide range of partial pressures up to 100 atm, (**b**) partial pressures relevant to applications. In (**b**) size of the symbols present the largest uncertainty in N_ads_. Symbols in panel (**b**) have the same meaning as in panel (**a**).

The number of CO_2_ molecules adsorbed (N_ads_) in the simulation cells S0–S4 is shown in Figure 2 as a function of the partial pressure of CO_2_. No normalization with respect to the adsorbent amount is carried out to represent the adsorption isotherms here, as the objective is to directly compare the amount adsorbed in different simulation cells (S0–S4). A wide range of adsorption isotherms with partial pressures up to 100 atm is shown in Figure 2a while the data at relatively low pressures relevant to applications are shown in Figure 2b. In Figure 2b, symbol size corresponds to the largest uncertainty in N_ads_. At high pressures (Figure 2a), the effect of inter-crystalline space on the adsorbed amount is monotonous—larger inter-crystalline space leads to higher adsorption. However, at the pressures relevant for applications (Figure 2b), this effect is non-monotonous. Initially, the adsorbed amount increases significantly adding inter-crystalline space from S0 to S2. Enhancement in the adsorption, however, is minimal from S2 to S3, and S4 even exhibits suppressed adsorption compared to S3. Based on these findings, we selected the inter-crystalline spacing of 5 Å (S2) to probe other effects as discussed in the next subsections.

**Figure 3:**
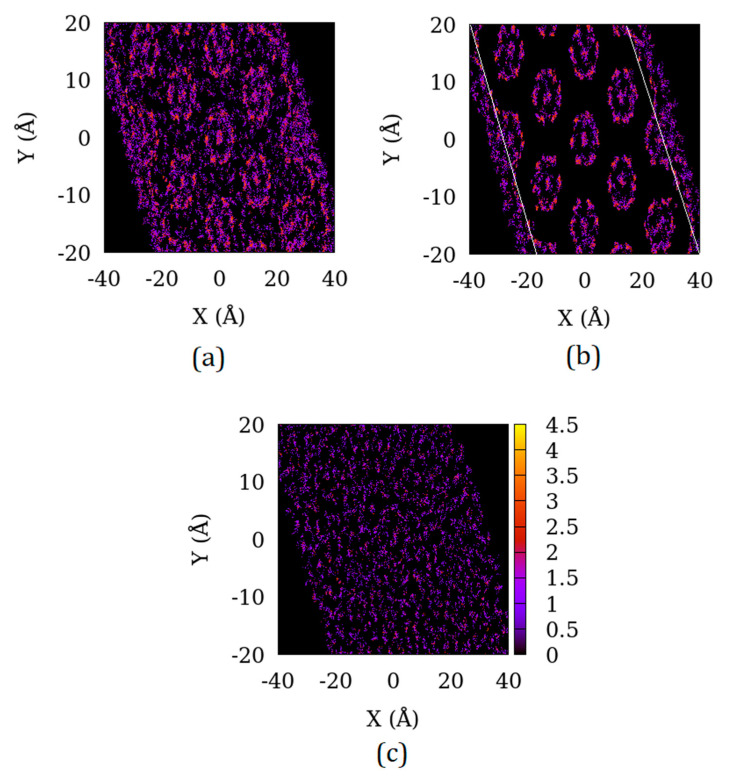
Distribution of the projection of center of mass of the adsorbed CO_2_ molecules in the X–Y plane of (**a**) S4 sample (**b**) S4 sample with the adsorbed molecules outside the substrate in the Z-direction excluded and (**c**) S4 sample with the adsorbed molecules inside the substrate in the Z-direction excluded.

In Figure 3, the distribution of the adsorbed CO_2_ molecules in the X–Y plane of the system S4 is shown. When all the adsorbed molecules are considered, (Figure 3a), a pattern of random distribution superimposed on a pattern of circular high adsorption points can be seen. When the molecules adsorbed in the inter-crystalline space outside the crystallite in the Z-direction are excluded, a pattern of strong adsorption in the crystallite pores (circular regions of high intensity in Figure 3b) are seen. In addition to the pores, the molecules adsorbed in the inter-crystalline space in the Y–Z plane can be seen making a random pattern (outside the crystallite boundary marked by white lines). The fact that the distribution of molecules in the inter-crystalline space is indeed random can be seen in Figure 3c where only molecules outside the crystallite in the Z-direction are included.

**Figure 4:**
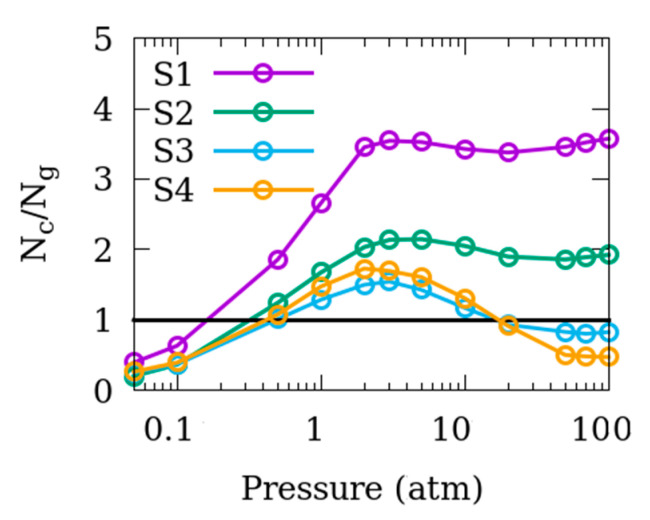
Ratio of the number of CO_2_ molecules adsorbed in the Mg-MOF-74 crystallites (N_c_) to the number of molecules adsorbed in the inter-crystalline space (N_g_) for the systems S1–S4.

Figure 4 shows the distribution of the adsorbed CO_2_ molecules in Mg-MOF-74 in terms of the ratio of the number of molecules adsorbed within the crystallite (N_c_) to those on the surface of the crystallite and in the inter-crystalline space (N_g_). At very low pressures, more molecules are adsorbed on the surface as compared to inside the crystallites. As pressure increases the pores within the crystallite start to fill while the amount of CO_2_ adsorbed on the crystallite surfaces increases relatively slowly thereby yielding (N_c_/N_g_) values larger than 1. This ratio increases consistently up to about 2 atm, after which it plateaus for S1 and S2 and decreases again for S3 and S4. The difference in the high pressure behavior between S1 and S2, and S3 and S4, is because of a smaller inter-crystalline space in the former two systems, which saturates with CO_2_ molecules at these pressures, whereas the latter two samples with a larger inter-crystalline space can accommodate a larger number of molecules thereby resulting in lower N_c_/N_g_ ratio at higher pressures.

### 3.2. Difference in the Surface Exposed

**Figure 5:**
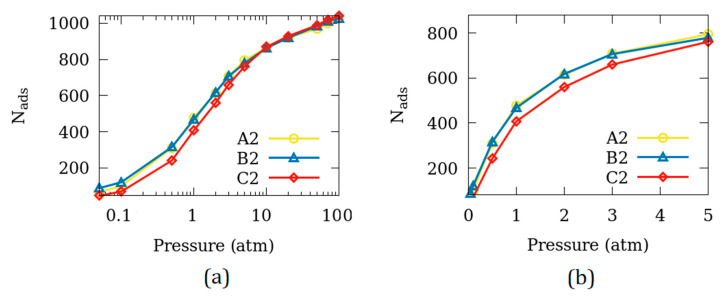
Effect of the surface exposed on the adsorbed amount over (**a**) full range of partial pressures and (**b**) the range of partial pressures relevant for applications. Only C2 sample shows a slight under-adsorption, probably due to the difference in the dimensions of the three surfaces. The uncertainty in N_ads_ is smaller than the symbols.

Figure 5 shows the comparison of adsorption amounts between the simulation cells A2, B2 and C2. These simulation cells differ in the surfaces that are exposed to the inter-crystalline space while the remaining surfaces remain un-exposed. Very little difference is observed in the behavior of the adsorption isotherms in A2 and B2 while the adsorption amounts at low pressures for C2 are slightly lower. We note that the surface area of C2 is slightly different from that of A2 and B2. The slight suppression in the amount of CO_2_ adsorbed on C2 compared to that on A2 and B2 may be a consequence of this difference in the surface areas.

### 3.3. Effects of Temperature

**Figure 6:**
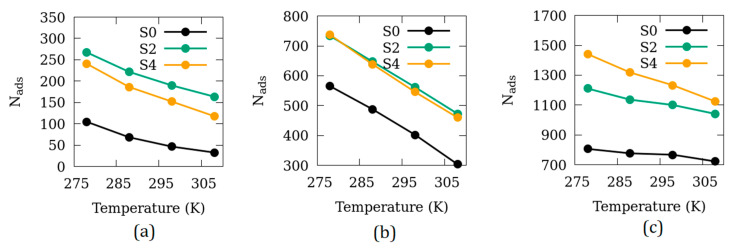
Number of adsorbed CO_2_ molecules in the systems S0, S2 and S4 as a function of temperature at (**a**) 0.1 atm; (**b**) 1 atm; (**c**) 10 atm.

To investigate if there exists any temperature dependence in the effects of inter-crystalline space on the adsorption of CO_2_ in Mg-MOF-74, we carried out more simulations at three pressure points at different temperatures. The results of these simulations are shown in Figure 6 in terms of N_ads_ as a function of temperature for the systems S0, S2, and S4. The temperature dependence of the adsorbed amount seems to be similar for the three systems at lower pressures. However, at high pressure, S4 adsorption exhibits a stronger temperature dependence (steeper slope for S4 in Figure 6c) compared to S0 and S2. This is probably due to the larger inter-crystalline void volume available in S4.

### 3.4. Effects of the Crystallite Size

**Figure 7:**
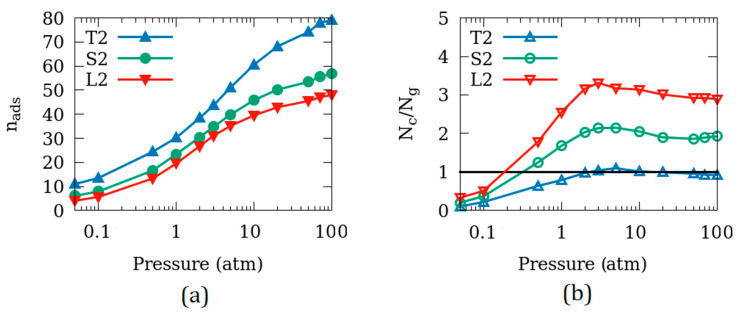
(**a**) Amount of CO_2_ molecules adsorbed per unit cell of Mg-MOF-74; (**b**) N_c_/N_g_ ratio of the CO_2_ molecules adsorbed in crystallites of different sizes.

Figure 7a shows the adsorption isotherms of CO_2_ in Mg-MOF-74 with different crystallite sizes–T2, S2, and L2–all with the same amount of inter-crystalline spacing. To make the comparison between crystallites of different sizes objective, the amount of adsorption has been normalized to one unit cell. Thus, number of CO_2_ molecules adsorbed per unit cell of Mg-MOF-74 (n_ads_) for the systems T2, S2, and L2 are shown in Figure 7a. The amount of adsorption per unit cell decreases as the crystallite size increases. Further, the difference between S2 and L2 (the larger two of the three sizes) is smaller than that between T2 and S2. This is probably because the surface to volume ratio of the crystallites increases strongly from T2 to S2 and relatively weakly from S2 to L2.

Figure 7b reveals that CO_2_ molecules are predominantly adsorbed on the crystallite surface of T2 at most pressures. The amount of inter-crystalline space available in T2 is larger than the pore space available within the crystallite for adsorption. As the surface to volume ratio of the crystallite decreases in S2 and L2, more and more CO_2_ molecules are adsorbed within the crystallite increasing the N_c_/N_g_ ratio beyond 1 for all but the lowest pressures. 

## 4. Discussion

The data reported in Figure 2 show that the amount of CO_2_ adsorbed in Mg-MOF-74 is significantly enhanced as inter-crystalline space is introduced between the crystallites. Further, at application-relevant low pressures, the adsorption amount increases as the extent of the inter-crystalline space is increased. This enhancement starts diminishing when increasing this space beyond 5 Å, while at high pressures, the adsorbed amount keeps increasing with the inter-crystalline spacing. This is because, at lower pressures, strong adsorption sites are needed for the CO_2_ molecules. These are available on the crystallite surface or in the crystallite pores. At an inter-crystalline space of 3 Å, the surface of the crystallite becomes exposed, making additional adsorption sites available, thereby resulting in an enhanced adsorption in S1 compared to S0. However, because the kinetic diameter of CO_2_ (3.3 nm) [38] is larger than this inter-crystalline space, adsorption on the surface is difficult and the enhancement in adsorbed amount is limited by geometrical restriction. As more inter-crystalline space larger than the kinetic diameter of CO_2_ is added in S2, the adsorption amount is significantly enhanced because of the relaxation of geometrical restriction. The inter-crystalline space is further widened to more than twice the kinetic diameter in S3. This means that the surfaces of two adjacent crystallites facing each other can each accommodate a single layer of CO_2_ and so the adsorption amount is increased again though marginally. However, further addition of inter-crystalline space does not enhance the amount of adsorption at low pressures because the adsorption sites available on the surface are saturated. At high pressures, the extra available space can accommodate more CO_2_ molecules in bulk-like region leading to an overall enhanced adsorption. This is also consistent with the fact that, for smaller inter-crystalline spacings, the adsorption amount is dominated by the CO_2_ molecules adsorbed within the crystallite pores whereas for larger inter-crystalline space and especially at high pressures, the adsorption is predominantly at the crystallite surface (see Figure 4).

Comparing the N_c_/N_g_ ratios in Figure 4 obtained for CO_2_ adsorption in Mg-MOF-74, with those reported earlier [17] for CO_2_ in silicalite, important and insightful differences are observed. In the case of silicalite, the adsorption of CO_2_ occurred predominantly on the surface of the crystallites at all pressures and irrespective of the extent of inter-crystalline space (N_c_/N_g_ < 1 for all cases, see Figure 3, reference [17]). In contrast, results in Figure 4 indicate more CO_2_ molecules are adsorbed in the crystallite pores compared to the surface at all but the lowest two pressures for S1 and S2. For S3 and S4, the ratio (N_c_/N_g_) is greater than 1 for partial pressures between 0.5 and 10 atm. This demonstrates that the crystallite pore adsorption of CO_2_ in Mg-MOF-74 is much stronger as compared to that in silicalite, revealing the superiority of Mg-MOF-74 as an adsorbent in CO_2_ storage applications compared to silicalite and other zeolites. This superiority has been attributed to the ionic character of the Mg-O bond in Mg-MOF-74 [39].

Temperature does not appear to significantly affect the role of inter-crystalline space in the adsorption of CO_2_ in Mg-MOF-74. The systems S0, S2, and S4 all exhibited similar trends with temperature of the adsorbed amount at 0.1 and 1.0 atm partial pressures. At a higher partial pressure of 10.0 bar, however, the adsorption amount in S4 shows stronger temperature dependence. This is because, in S4 at this pressure, more CO_2_ molecules are weakly adsorbed close to the crystallite surface in the inter-crystalline space. While molecules adsorbed strongly within the crystallite pores and crystallite surface are affected by temperature in the same way as those in the systems S0 and S2, the additional weakly adsorbed molecules in S4 in the inter-crystalline space away from the crystallite surface are affected more by the temperature and are easily desorbed at higher temperatures. This leads to a stronger temperature dependence of adsorption in S4.

The dependence of adsorption amounts on the size of the crystallites shown in Figure 7 is consistent with the dependence observed in ref. [17] of the amount of CO_2_ adsorbed in/on the crystallites of silicalite. This dependence on the crystallite size is probably dominated by the geometric effects of the surface to volume ratio rather than the chemistry of the substrate. As the introduction of inter-crystalline space enhances the adsorbed amount by making the crystallite surface available for adsorption, in addition to the crystallite pores, this enhancement can be expected to be proportional to the size of the exposed surface. As the size of the crystallite gets larger, the surface to volume ratio decreases and hence the adsorbed amount decreases. This suggests that, for applications in CO_2_ storage, the ideal Mg-MOF-74 substrate should have small crystallite size. 

The dependence of adsorption amount of CO_2_ on the surface area has been experimentally investigated for Mg-MOF-74 by Yao et al. [40]. They synthesized Mg-MOF-74 materials of three different morphologies and sizes (200 nm–4 μm). The amount of adsorption was found to be higher for samples with higher surface area. This is consistent with our results reported here. In a recent work, Campbell and Tokay [41] have also shown that the crystal size of Mg-MOF-74 can be controlled by varying the fraction of ethanol and water in the reaction solution relative to dimethyl formamide. By varying this composition, they obtained smaller crystallites with sizes varying between 8 nm to 50 nm. It is also possible to make hierarchical MOF with multi-porosity introduced in the system [42]. These hierarchical systems, however, have crystallites arranged in an ordered fashion along with mesoporous or even macroporous formations. As found in this work, inter-crystalline spaces larger than a few angstroms need not necessarily result in an enhancement in adsorption. Conversely, the trends of adsorption capacity with the crystallite size suggest that a single unit cell of MOF might be the best for adsorption. This single unit cell would represent a metal–organic polyhedral (MOP) [43]. However, limitations of computational accuracy forbid the calculation of adsorption in a single unit cell. With a potential cut-off of 14 Å as used here, the smallest simulation cell dimension cannot be smaller than 28 Å while the unit cell dimension of Mg-MOF without any inter-crystalline space is 26.136 × 26.136 × 6.942 Å^3^. Therefore, it is not possible to verify with GCMC if the MOP analogue of Mg-MOF-74 can show better adsorption capacities for CO_2_. While we are not aware of an experimental study on the adsorption of CO_2_ in MOP analogue of Mg-MOF-74, we note here the work by Lorzing et al. [44] on gas adsorption in metal–organic polyhedra. In this work, the authors observe that MOP have significantly lower surface areas compared to their MOF analogues. This would imply that MOP analogue of Mg-MOF-74 might not achieve significantly higher adsorption of CO_2_. However, further experimental investigations are needed to verify this. Inter-crystalline spaces larger than twice the kinetic diameter of CO_2_ is enough to enhance the adsorption. Thus, an ideal Mg-MOF-74 adsorbent would have small size crystallites separated by inter-crystalline space of approximately twice the kinetic diameter of the adsorbed fluid. 

## 5. Conclusions

We have studied the effects of inter-crystalline space on the adsorption of CO_2_ in Mg-MOF-74. The inter-crystalline spacing is found to enhance adsorption by making the adsorption sites at the crystallite surfaces available for adsorption. At pressures relevant to capture applications, increasing the inter-crystalline spacing up to twice the kinetic diameter of CO_2_ results in higher adsorption while further increasing this space does not lead to any significant increase in the adsorption amounts. Larger crystallite sizes suppress adsorption amounts due to a smaller surface to volume ratio in large crystallites. The size of the inter-crystalline space relative to the kinetic diameter of the adsorbed fluid and the surface to volume ratio of the crystallite size of the adsorbents thus play an important role in determining the adsorption amounts. The results of this study help guide our strategies for the tailoring of Mg-MOF-74 samples for high performance as CO_2_ adsorbents. The ideal Mg-MOF-74 sample for CO_2_ storage applications should have smaller crystallites separated from each other with an inter-crystalline space of roughly twice the kinetic diameter of CO_2_.

## Figures and Tables

**Table 1:** Summary of simulation cells used in the study.

Name of the Simulation Cell (System)	Number of Unit Cells in the Simulation Cell	Surfaces Exposed to Empty Space (Side Exposed*)	Size of the Empty Space (2× Space on Each Surface, in Å)
S0	2 × 2 × 6	0	0
S1	2 × 2 × 6	6	3.0
S2	2 × 2 × 6	6	5.0
S3	2 × 2 × 6	6	10.0
S4	2 × 2 × 6	6	15.0
A2	2 × 2 × 6	2 (A*)	5.0
B2	2 × 2 × 6	2 (B*)	5.0
C2	2 × 2 × 6	2 (C*)	5.0
T2	1 × 1 × 4	6	5.0
L2	3 × 3 × 9	6	5.0
